# Case Study of the Effect of Precoating on the Decarburization of the Surface Layer of Forged Parts during the Hot Die Forging Process

**DOI:** 10.3390/ma14020422

**Published:** 2021-01-16

**Authors:** Paweł Widomski, Maciej Zwierzchowski, Artur Barełkowski, Mateusz Tympalski

**Affiliations:** Department of Metal Forming, Welding and Metrology, Faculty of Mechanical Engineering, Wroclaw University of Science and Technology, 50-371 Wrocław, Poland; maciej.zwierzchowski@pwr.edu.pl (M.Z.); artur.barelkowski@pwr.edu.pl (A.B.); mateusz.tympal@gmail.com (M.T.)

**Keywords:** decarburization, precoating, hot forging

## Abstract

This paper aims to evaluate the effect of pre-coating of forged parts on decarburization in the die forging process. The studies consisted of three stages. In the first instance, different coatings were tested under laboratory conditions by heating steel samples to the temperature of 1200 °C for over five minutes to model the preheating conditions of the induction. Next, testing continued in a commercial forging stand where we tested the effects of different coatings on the rods decarburization during the induction heating process, usually performed before forging. Once completed testing, the measurements and observations of the decarbonized layer were made. The third stage involved analysis of the decarburization of the forged parts after forging. The forged parts were made using precoating of pre-forging elements; pieces cut off a metal rod. Based on tests results, the possibility of using this solution in the technique of industrial hot forging was evaluated. The results of laboratory tests have confirmed that lubrication of metal pieces is sufficient, as well as proved it to be effective in reducing decarburization of the surface layer. Research works conducted in an induction heater showed differences in decarburization depending on a substance and concentration of lubricants that were used. These differences become more apparent when observing the surface layer of the forged parts. Results indicate that decarburization may be reduced to a minimum when we use Bonderite product in a concentration of 66% and 50%. Another lubricant, Berulit 913, may also be used. However, due to burning graphite in high temperatures, reduction of decarburization goes only as far as half of the thickness of the decarbonized layer. Condursal has no significant effect; nevertheless, it protects over the induction heating stage.

## 1. Introduction

Oxidation processes are very problematic. Depending on heating parameters, even up to 3% of steel weight might be turned into a scale when heated, so it is a waste. Every year, a significant amount of steel parts production in Europe is challenged owing to decarburization they contain [1]. Given the vast scale of this manufacture, it can be estimated how much material is wasted and acknowledged that losing properties in the surface layer as a result of decarburization plays a vital role. Properties reduction is detrimental to carbon steel in particular; the steel which properties depend on carbon content. Oxidation and decarburization of steel are carried out simultaneously and occur over heating in an oxidizing atmosphere [2]. Decarburization in higher temperatures leads to 38 constant carbon diffusion from a surface layer towards the surface; in result, the surface layer of material is decarbonized. Decarburization decreases the layer strength, and that brings a drop in performance parameters [3]. 

Many attempts have been made to reduce both phenomena of oxidation and decarburization of steel. Occasionally, the heating conditions are defined to make steel oxidation proceed quicker than carbon diffusion and decarburization [4]. Using a protective atmosphere with a reduced amount of reactive oxygen is common. Furnace atmospheres can be: Oxidizing, neutral or reducing depending on thermal processing methods. The protective atmosphere consists of the following gases: Methane, ethane, propane, argon, hydrogen, helium and nitrogen; alternatively, it may be a vacuum [5]. 

Traditionally produced atmospheres usually develop within the constant intensity of the flow and combination of gases like EXO and Endo (for instance, a mixture of the air and pure propane in accurate proportions as illustrated in Table 1). Some extra control over this process may also be implemented by using atmospheres obtained from nitrogen dilution (Table 2). Into the last category fall mixed atmospheres (Table 3), based on nitrogen and a controlled number of additives. These atmospheres may be of satisfying and low quality. To fully utilize the advantages of these atmospheres, often, other interactions on the side of the operator or control instrumentation must be employed.

One of the most popular protective atmospheres is the nitrogen atmosphere. It is obtained from either an endothermic generator or through combining nitrogen and hydrogen. The advantage of this atmosphere is, among other things, not complicated and relatively inexpensive instrumentation, easily accessible material and a broad spectrum of usages. The nitrogen-based atmosphere with a specific carbon capacity may be handily formed by adding a controlled amount of H_2_O, regardless of the carbon measure in the material itself. The atmosphere is safe for the thermal process since CO, and H_2_ fractions are present on a minuscule scale in this atmosphere.

Some instructions suggest to use atmospheres to heat high-carbon steel. The greater content of combustible material (high content of CO and H_2_, low content of CO_2_ and H_2_O), the higher tolerance on impurities from leakages as well as oxides on a workpiece. Results of the heating process also relate to properties of a surface of the material which goes into the furnace. Spotless surface means better heating results [6]. 

A popular way of producing an atmosphere, which prevents decarburization, is pouring a neutral carbon into a chamber over the annealing operation to protect high-carbon and tool steel from oxidation and decarburization. This carbon binds oxygen throughout the whole process. Unfortunately, introducing a protective atmosphere is not always possible due to some stipulations referred to, the specification of operations carried out in the open space and due to economic reasons. In this case, some protective coating, which will separate the surface from a reactive oxygen atmosphere, may be used. The surface layer of material should be covered with pastes and liquids of different composition. The most popular techniques of substance application are immersing, brush application, spaying and lubrication. Some agents allow a complete surface coverage others give only a thin layer when not much of a lubricant is used. The main problem with using protective agents is high temperature. The steel must be heated before forging. 

In some cases, it may even come up to 1250 °C, and thus, significantly limit the range of substance which may be used. Various water-based coverages are available (containing mainly silicates) as well as these based on organic solvents. These might be formulas based on dolomite, bauxite and silicon carbides which form when mixing these three elements in different proportions. The solution is diluted with water; it gives better adhesiveness, that allows painting and spraying. Thanks to adding a binding agent (citric acid), adhesion to the surface after drying out improves. The central agent of this mixture is dolomite, the content of which is around 50–70%. Dolomite contains mainly calcium carbonate and magnesium carbonate, and since magnesium oxides have a high melting point, they might be used as a base for the coating applied. About 20% to 40% SiC might be found in the mixture. Silicon carbide powder oxidizes gradually and turns into a protective coating of cristobalite—SiO_2_, which provides an excellent barrier for the oxygen diffusing from the atmosphere. The layer of SiO_2_ does not derive from SiO_2_ present in dolomite, but rather forms by SiC oxidation. During SiC oxidation, graphite is formed as well; this helps to reduce the harmful effects of the atmosphere, even at high temperatures. This agent may also contain from 20% to 40% of bauxite. Bauxite reaction depends on its quantum. Bauxite decomposes at high temperatures and reacts with iron and dolomite. Products of this reaction are SiO_2_, FeO·Al_2_O_3_, α-Al_2_O_3_ and spinel (MgAl_2_O_4_); with no oxygen affinity. Thus, the thickness of the decarburized layer is notably reduced, along with an increase in steel oxidation resistance [7,8,9]. 

Another group of protectives consists of pastes based on zircon. They are equally high heat resistant, up to 1800 °C, and thermal shocks resistant [6]. Another method of protecting a surface from decarburization and oxidation over the heating process is using ATP (Adenosine Triphosphate) coatings. These are advanced glass or ceramic coatings, water-based, safe for use and highly economical when it comes to protecting metal surfaces in high temperatures. These agents may endure heat up to 2400 °C. Their formula aims to limit gases diffusion from and to metal. They are applied before heating employed in rolling, forging, hot stamping and other thermal finishing treatments. The main objective is to prevent metal and alloys from oxidation in oxidizing atmospheres, heated for a more extended period. ATP coatings may be applied on tool steel, rustless metal, nickel alloys, carbon steel, zircon, titanium and molybdenum alloys. The cost-effectiveness of the coating comes from the fact that it delays the creation of oxides layer, reduces surface defects, minimizes the demand for finishing operations, and reduces material failure by improving product quality.

The product should be applied to a chemically cleaned area. The thickness of the layer is determined by a kind of metal, time and heating temperature. Water-based coatings must be dry before being placed in a furnace. They may be put on metal heated up to 150–200 °C [10]. The shielding barrier may also be created on the steel surface before heating by employing compounds CrR_3_ and AlR_3_. It is both an economical and eco-friendly method. Deposition of oxycarbide and oxide phases, on the top part of the heated elements, produces a barrier which stops oxygen from deep material penetration and does not allow the gas phase to come into contact with the steel. It develops a thin film with FeO, on the surface, which gives a base for a protective layer containing C_2_O_3_ and Al_2_O_3_. Oxygen diffusion through the barrier is significantly slower. On this account, trivalent iron phases Fe_3_O_4_ and Fe_2_O_3_ do not form under the shielding layer, and the increase of FeO phase is remarkably restricted, as it may be penetrated only by iron ions over cation subnet. The tempo of this diffusion is a rate slower than a pace of oxygen ions diffusion, through phases of Fe_3_O_4_ and Fe_2_O_3_. On the surface of the protective barrier, the Fe_2_O_3_ film also builds up slowly due to restricted diffusion of Fe^2+^ ions through this barrier. The total thickness of oxides on the metal surface in the final stage should not exceed 2–3 ηm even after extensive heating up to 1500 °C. This type of covering is not structural and does not affect the mechanical properties of a metal. The layer thickness is within 20–50 nm. When the coating is based on chromium oxide, metal (chromium) only infiltrates steel to a depth of 20–40 nm. Additionally, decarburization of the surface layer is notably slower due to the moderated rate of carbon diffusion from the deeper metal layers. On account of this process coatings produced from chromium, and aluminum compounds may be widely used in metallurgical, machinery, aviation and space industry as well as in forging and rolling.

Another group of agents, essential for this research, are graphite-based substances. They come as a suspension of graphite particles in the water. Surfactants and binders must be added to these solutions to facilitate application and spreading of thin layers of a lubricant. Mixtures containing graphite are popular in the metal processing industry by virtue of cost-effectiveness and graphite thermal resistance. Lubricant properties do not originate from the graphite crystalline structure alone. They are also a result of the steam deposits which were absorbed, giving a low cohesion surface. Steam is essential for a lubricant; therefore, graphite is not usually useful in a vacuum. In an oxidizing atmosphere, these substances are useful in high temperatures. An agent’s ability to minimize the adverse effects of oxidation lies in the quality of graphite, particles diameter and additional impurities, which may accelerate oxidation. Graphite as such has low thermal conductivity. These products are usually spread on by spraying, immersion or painting. One of the problems concerning graphite-based products is frequent thinness in the film, which results in lower productivity. Advantages of graphite water suspensions are, among other, excellent lubrication, reduced wear of forging tools, a wide range of graphite grain size (up to 50 μm) and adequate coverage. Additionally, they do not contain heavy metals, are easy to dilute, cost-effective and inflammable; and do not include ammonia [11].

Over the above methods of using atmospheres and coverings in order to reduce decarburization, heat exposure may be downsized too. It may be achieved by optimization of the heating time, improving the economy of induction heaters and accelerating forging process thanks to automatization. Apart from heating parameters, the material as such is also worth attention. Increasing or downsizing of some alloy elements should be taken into consideration, too. For instance, chrome in high temperatures produces stable carbides reducing a stable austenite range and in consequence reduces the amount of carbon that could diffuse into the steel surface. The right manipulation of alloy’s elements may significantly limit the unwanted effects of oxidation and decarburization. Moreover, the quality of the surface may be improved, which will impact its chemical activity. Imperfections on the surface amplify relative parameters of the sample and cause quicker oxidation, as well as change the proportions of the decarburized surface. It is also profitable to remove all cracks and barbs, from the surface, through which the oxygen can easily penetrate deeper layers. Polishing helps to spread the decarburized layer and oxides evenly. In this study, peeled rods were used. Peeling is an operation of removing a thin layer from a steel rod by mechanical means.

Finally, the approach on several different levels is possible when it comes to preventing decarburization and oxidation in thermal conditions. This paper presents some selected methods which employ coverings based on organic solvents and water suspensions of graphite [12]. These substances have not been used in thermal forging so far, for it is not popular to use lubricants for steel forged parts over the warming process. Only some attempts of using coating during warm forging are known; where on account of initial graphitization, the decarburization is reduced, and there is less of mill scale [13]. We also know about experiments attempting to define the correlation between different coatings and mill scale quantum in the process of first warming and forging [14].

The dilemma of decarburization and oxidation of the surface layer of the forged and pre-forged parts and the lack of satisfactory technological solutions prompt further research.

It is known that some smithies in India (Bharat Forge) and Germany (Henkel) have also attempted to find a solution in this matter [15]. Other European forge plants are seeking for best answers too. Therefore, the demand for further research is still current. The industry expects that technology should be efficient and cost-effective at the same time, as well as possible to be implemented in the manufactures. Having regard to previously mentioned, this research focused on inexpensive products, easily accessible and useful for the application. The agents were tested in order to find ways of practical application, define optimal concentration and verify protection effectiveness under laboratory and industrial conditions. 

## 2. Materials and Methods 

Research devoted to exploring the correlation between protective coatings and decarburization were conducted on the field of hot die forging of the yoke type forging. It is an element of the steering system. Yoke type forging is produced in the forge Kuźnia Jawor over multi-operational hot die forging process in a press of 1300 T pressure. The initial material was a steel rod C45 diameter 35 mm prepared by peeling. The rod was cut into pieces of 132 mm which then went under the induction heating process in the temperature of 1150–1180 °C [16]. This lasted for approximately 3 min, depending on the heater efficiency. The effectiveness of heating the rods in the induction heater was assessed by numerical modeling, in which the dependence of temperature on time inside and on the rod surface was determined. The heater sorts hot rods and according to temperature qualifies as suitable for forging. Next, the rods were transported over the conveyor to the robotic stand where they were manipulated by a robot over the forging process. The whole procedure was operated by robots and manipulators; it was automated, too. When considering decarburization, the most important aspect are the procedures when a forged part is heated in high temperatures. When 1100 °C is exceeded, carbon diffusion accelerates. What is more, no protective atmospheres may be employed during manipulation, forging and cooling. The heating transaction could be carried out in a protective atmosphere; however, this is not in practice because of economic reasons. In order to debase oxygen influence, it is possible to downsize the induction heating time, manipulation time and apply a thin layer of lubricants on the surface. This research proposes three protective coatings to reduce decarburization:

1. Berulit 913 (producer: Carl Bechem GmBH, Gardelegen-Mieste, Germany)
2. Bonderite L-FG FB 685 (Producer: Henkel, Düsseldorf, Germany)
3. Condursal Z1100 (Producer: The Duffy Company, Palatine, IL, USA)

Berulit 913 is a graphite-based lubricant, used in a semi-hot forging and hot rolling in up to 950 °C [17]. It has a fine lubrication quality. It prevents decarburization and scale formation in hot temperatures. It may be used in operation for steel and aluminum. It is useful as a concentrate as well as dissolved in the water in proportion 1:8. It is black and does not contain oil. In the room temperature (20 °C) its density is 1.1 g/cm^3^. Should not be sprayed. Painting and immersion are recommended.

Bonderite L-FG FB 685 Acheson is based on graphite water solution, designed for hot die forging of ferritic material. It contains neutral graphite, grounded down to particles to improve lubrication. The following methods of application are recommended: Spraying, painting and immersion. May be used in the form of a concentrate or dissolved in the water. No specification of maximum temperature for usage has been given by the manufacturer.

Condursal helps to stave off oxidation and decarburization of both steel and stainless steel, thus preventing overheating in oxidizing atmospheres. Before application the material must be cleaned, free from oils, grease or other contaminants. Any kind of impurity may stop Condursal from the total covering of the sample and in effect cause uneven decarburization and oxidation during heating. Condursal may be applied by painting or immersion. Only a layer of 15 µm thickness is needed for optimum protection. The manufacturer recommends a temperature of 1100 °C to optimize effects. The residues, once the heating is finished, may be removed using a steel brush.

The research was carried out under laboratory conditions and examined forging parts in the course of forging. The experiment consists of three stages, conducted in order to undeniably confirm the influence these substances have on decarburization of pre-forged and forged parts.

### 2.1. Laboratory Test Conducted in a Furnace without a Protective Atmosphere in Conditions Modelling Induction Heating

To define heating time corresponding to induction heating, a few trials were carried out. First the numerical modelling of heating process was performed to obtain real temperature of rod inside and on the surface during induction heating. Another test show that within 5 min, in a furnace of 1200 °C decarburization similar to an industrial one takes place. Therefore, steel samples C45 were coated by three different products of various concentrations, and after that, they were heated for 5 min in a furnace without a protective atmosphere in a temperature 1200 °C. They were then cooled in the air, and decarburization of the surface layer was examined. In order to evaluate the effect of the coatings on the decarburization of the surface layer the microstructure analysis was performed. Samples were cut perpendicular to the bar axis, the cross section was polished and pre-etched with Nital 3%. The microstructure observations were done on the Olympus GX-51 optical microscope (Olympus, Tokyo, Japan).

### 2.2. Laboratory Tests Carried out in the Industrial Environment Using an Induction Heater Popular in Hot Die Forging

A 400 kW induction heater (Termetal, Graniczna, Poland) was used for the tests, designed for rods of 35–70 mm diameter. In these tests, coatings on steel rods, diameters 35, 45 and 70 mm, were tried. Three protective substances in diverse concentration, diluted with water were tested:

1. Berulit—concentration; 100%, 66%, 50% and 33%
2. Bonderite—concentration; 100%, 66% and 50%
3. Condursal—concentration; 100%

Pieces of metal rods were pre-coated by immersion then dried in a vertical position. After drying out, rods were heated in an induction heater in which they were moving towards the exit, where the temperature measurement was taken. The temperature was 1150–1180 °C which meant that they were fit for forging. Nevertheless, after heating the rods were placed for cooling in the air. Finally, decarburization was analyzed. It showed that among all rod’s diameters, those with the least diameter were decarburized the most, that is, rods of 35 mm diameters. Therefore, based on these rods, the analysis of the coatings and decarburization correlation was carried out. Samples similarly to the bars from previous section were cut perpendicular to the bar axis, the cross section was polished and pre-etched with Nital 3%. The microstructure observations were done on the Olympus GX-51 optical microscope.

### 2.3. Industrial Tests on Forged Parts in the Industrial Hot Forging Process

Rods were covered in a protective substance then heated in induction, placed for hot die forging and cooled in the air. In all the forged parts the surface layers were proved with a view to decarburization. Three protective substances in diverse concentration, diluted with water were tested:

1. Berulit—concentration; 100%, 50% and 33%
2. Bonderite—concentration; 66% and 50%
3. Condursal—concentration; 100%

The above concentration selection is a result of the second stage of this research, where we stumbled upon achieving a complete coating for the substances containing too much of a base product. When they were too dense, the lubricants created too thick a layer, which peeled off and gave no protection. In result, the above concentration was determined.

In order to assess the effect of the coatings on the decarburization of the surface layer the thorough analysis was performed. Testing was carried out after the forging process, samples were cut out of the forgings, polished and pre-etched with Nital 3%. The analysis included observations of the microstructure on the Olympus GX-51 optical microscope and the Tescan Vega 3 scanning microscope (Oxford Instruments, Oxford, UK).

## 3. Discussion and Results

The tests were focused mainly on applying coatings over the hot die forging. In the first stage, the correlation between heat resistance of lubricants was tested alongside their influence on decarburization in the environment modelling the real one. On this basis, the concentration of lubricants was redefined, which was then tested during the real hot die forging in the next two stages. After that, the forged parts were examined in the perspective of lubricants and decarburization.

### 3.1. Results of Laboratory Tests Conducted in the Environment Mapping Induction Heating Process

**Figure 1:**
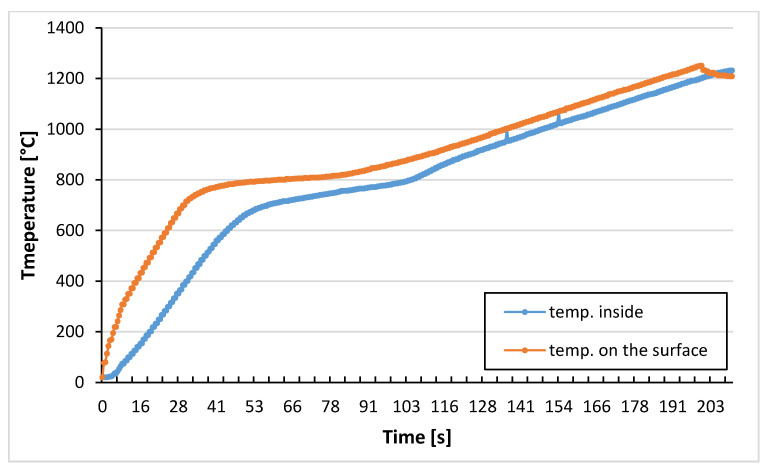
The temperature of rod during the process of induction heating.

Firstly, the numerical model of induction heating process was built to determine the temperature inside and on the surface of steel rods during the process of induction heating. The model of heating was based on the assumptions of several parameters taken from the literature and database (heat transfer coefficient in contact and with air, outside temperature, humidity, etc.). The idea behind the modelling was to find out the real time of exposure to high temperatures over 800 °C and more. The result of modelling was presented in Figure 1. The values obtained in the model were confirmed by the actual heating time in real process and the boundary values of temperature at the end and beginning of the process.

The exposure time in critical temperature under 800 °C is reduced to about 120 s (measured on the surface) thanks to the special heating strategy in comparison with typical induction heaters. This period of time is the most important, because the diffusion of carbon and reactions with oxygen increase exponentially. Therefore, this critical period of time should be shortened as it is possible to avoid decarburization.

**Figure 2:**
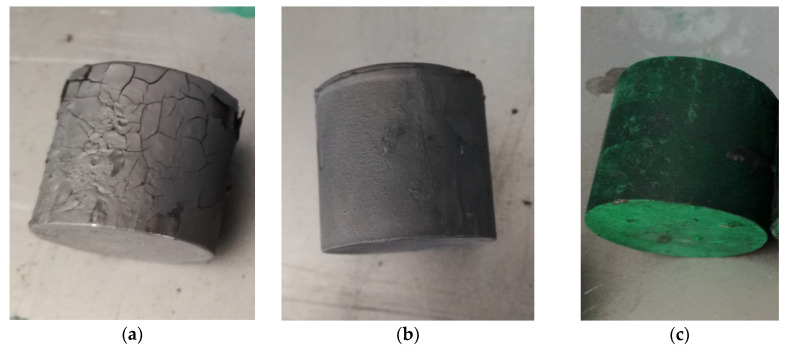
Pictures of the pre-forged parts after lubrication: (**a**) Berulit, (**b**) Bonderite and (**c**) Condursal.

To model the influence of heat on the coatings, Authors proposed laboratory conditions by heating in non-atmospheric furnace. In such conditions the possibility of covering by protective layers was tested. In Figure 2 pictures of rods coming from a steel bar pre-coated with protective layers are presented.

**Figure 3:**
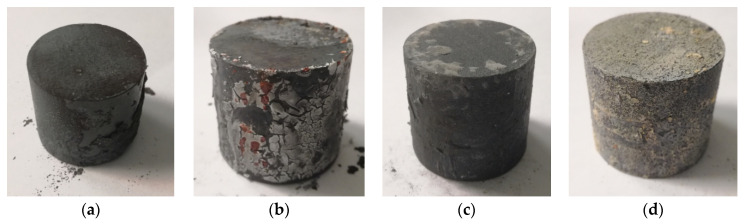
Pictures of the samples after heating T_a_ = 1200 °C in the time of t_a_ = 5 min after cooling; (**a**) no coating on, (**b**) Berulite coating, (**c**) Bonderite coating and (**d**) Condursal coating.

The differences in the lubrication were observed. Berulit turned out to be too dense; therefore, it built up a layer which is too thick and cracked. It is advised to dilute the substance, but the manufacturers do not provide the optimal proportions. Thus, in the next part of the research, an attempt was made to use Berulit and Bonderite in different concentrations, diluted with water. Throughout heating, the material dries, in result, in high temperatures graphite oxidation may occur. When this happens, it loses its protective properties. Figure 3 depicts the surfaces of the samples after heating and cooling, where we may observe lubricants residues.

Layers in the above pictures are burned, cracked and in some spots even wholly removed off the surface. However, it is presumed that the surface layer was damaged in the last stage of heating when the temperature was over 1000 °C. Therefore, up to this point, the lubricant was protecting the surface, and this should reduce decarburization. Figure 3 shows the result of microstructure observation, which confirmed the expected differences in the thickness of the decarburized layer. Most of the cross-sections show pearlite structure with the ferrite on the grains. It is typical for C45 steel which is slowly cooled. By the top layer of the bar, in two cases, light grains of ferrite, with a bit of pearlite, may be observed. This amount of ferrite indicates where the process of carbon reduction in the steel occurred.

**Figure 4:**
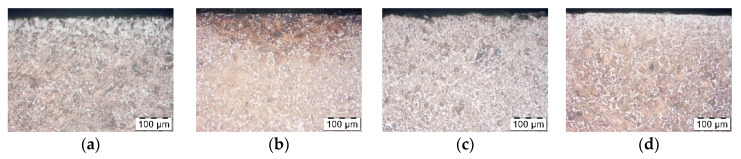
Microstructure in the surface layer of steel C45 after 5 min of heating in temp 1200 °C: (**a**) no coating, (**b**) Berulit, (**c**) Bonderite and (**d**) Condursal.

The researchers have confirmed unquestionably the reduction of decarburization owing to graphite-based lubricants: Berulit (Figure 4b) and Bonderite (Figure 4c). Condursal (Figure 4d), despite high-temperature resistance, which was expected, was only slightly affected by the thickness of the decarburized layer. The difference in the size of austenite grains, visible thanks to ferrite structure at the boundaries of pearlite grains, are caused by material recrystallization after cutting off pieces. The surface layer of rods is, in some parts, cold-pressed.

### 3.2. Results of the Study on the Effectiveness of the Protective Coating in Preventing Decarburization over the Induction Heating

**Figure 5:**
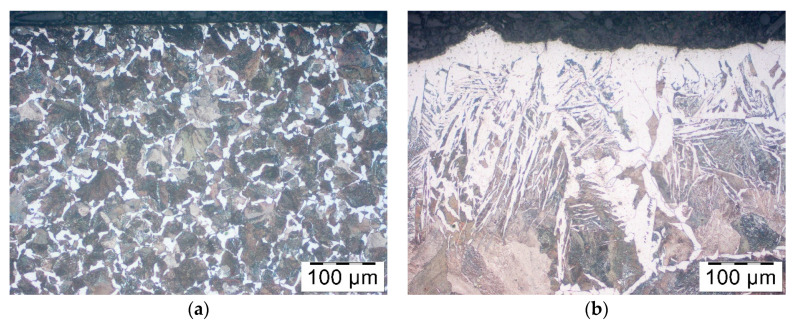
Microstructure of the surface layer of the steel bar C45: (**a**) when delivered and (**b**) after heated for forging and cooling in the air.

The study tested protective coatings, in correlation to decarburization of the steel bars C45, during induction heating and was carried out using pre-forged parts utilized in forging. Some of the analysis of the microstructure are presented in the pictures. In the first instance, the influence of the atmosphere on the sample with no coating was described. After heating a pre-forged part to the forging temperature and cooling in the air, the decarburization is visible as a dense ferrite structure (Figure 5b), which in some parts goes as deep as to approximately 250 µm. 

For comparison, a metallographic order of the sample cut from the rod before heating was performed (Figure 5a). The rod was delivered to the smithy without any prior thermal proceeding, after hot rolling and peeling. This is confirmed by a homogeneous cross-section of the microstructure, which consisted of ferrite and pearlite with the advantage of the latter. Decarburization was not observed at the surface, which indeed took place during the hot rolling process, since the entire top layer with the mill scale was removed over the peeling operation. When comparing both pictures, a large amount of grain spread was noticeable; this occurred during the warming and cooling of the pre-forged parts.

**Figure 6:**
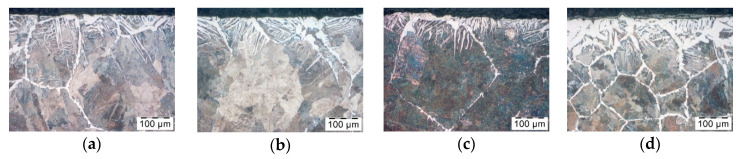
Microstructure of the surface layer of a steel bar C45 after induction heating, coated with Berulit—concentrations of (**a**) 100%, (**b**) 66%, (**c**) 50% and (**d**) 33%.

In the next stage, decarburization after using Berulit was tested; the concentrations were 100%, 66%, 50% and 33%. Figure 6 shows the results. All pictures show a structure of ferrite which becomes denser by the surface layer of the pre-forged part. In the Figure 6a–c, the size and density of the structure is comparable, whereas, in Figure 6d, the structure is much denser and goes more in-depth in the rod. This means that the use of the Berulit is beneficial on the condition that the concentration is no less than 50%.

**Figure 7:**
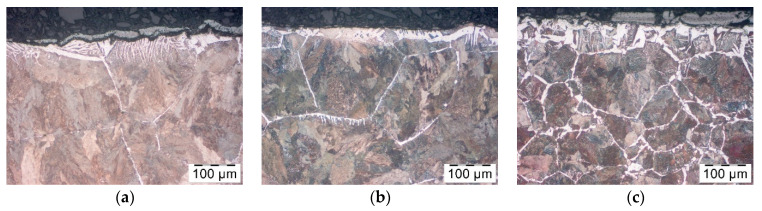
Microstructure of the surface layer of the steel bar C45 after induction heating with Bonderite coating—concentrations (**a**) 100%, (**b**) 66% and (**c**) 50%.

Decarburization subsequent to Bonderite usage was observed- the concentration of the substance was 100%, 66% and 50%. Figure 7 shows the results. We may see that in this case, ferrite grains do not occur in high density by the top layer and do not penetrate deep into the bar, compared to the former product. We observe higher density when the concentration of 50% is employed.

**Figure 8:**
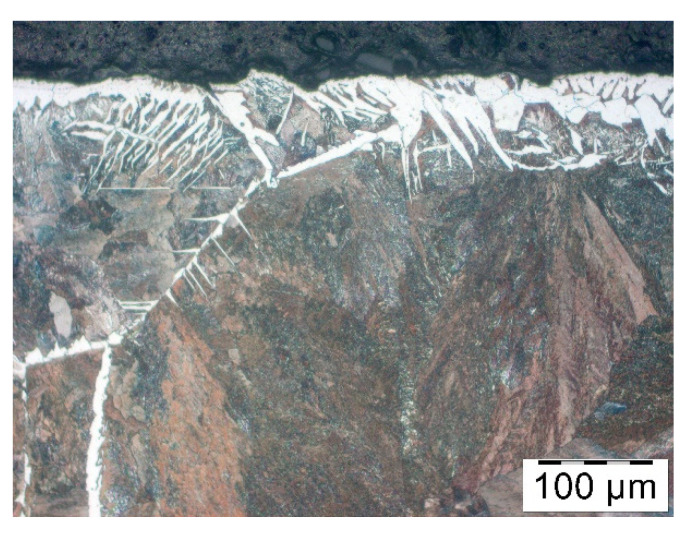
Microstructure of the surface layer of the steel bar C45 after induction heating, coated in Condursal—concentration 100%.

Lastly, decarburization after using a protective coating of Condursal was observed—concentration 100%. Only the concentration of 100% was tested due to low viscosity and density, which prevents it from sticking to the surface. Figure 8 illustrates that the surface layer was decarburized, even though the product was not diluted. The density of ferrite structure and how deep it goes was comparable only with the lowest concentration rates of other products.

### 3.3. The Results of Testing Forged Parts over Forging

**Figure 9:**
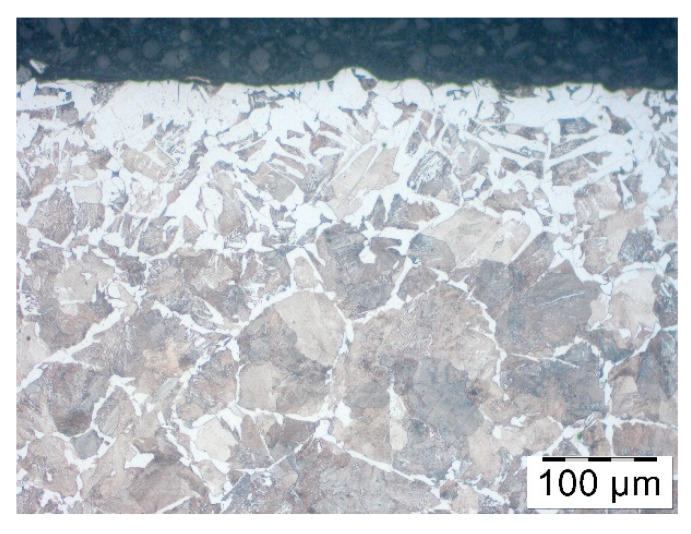
Microstructure in the top layer of the forged part after hot forging and cooling—with no lubrication.

In the third stage, the research on the ready-made forging was carried out. After coating the pre-forged parts were heated to the temperature of the forging and then forged over the hot forging process in three steps: Flattening, pre-forging and ready-forging. The last operation consisted of cutting the scrap. After that, the forged parts were dried in the air. The analysis of the microstructure was made. Initially, the change in the microstructure of the forged part was observed when no coating was applied (Figure 9). Moreover, the diameter of the grains were smaller as well. These changes were possible due to deformation in the heating procedure, which in the course of recrystallization caused fragmentation of the grains.

**Figure 10:**
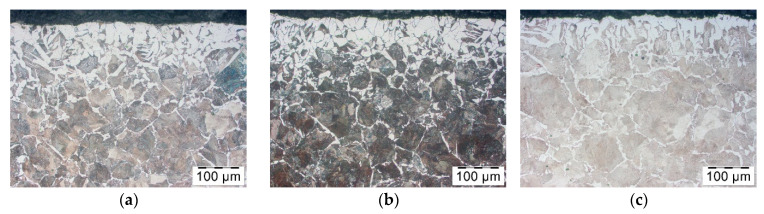
Microstructure in the surface layer of the forged part after hot forging and cooling—pre-coating in Berulite, in the concentration of (**a**) 100%, (**b**) 50% and (**c**) 33%.

Next, the decarburization of the surface layer of the forged parts after Berulite application was examined. The Berulite concentration was 100%, 50% and 33%. The three concentrations showed the same results as in the case of not using any coating (Figure 10). Taking into consideration the penetration caused by ferrite, the microstructure of the part where the 33% concentration was applied seems to be the most advantageous, what is doubtful as in Figure 6 the effect of coverage was the least at this concentration. Therefore, it should be assumed that the effect of Berulite coating on decarburization observed after the forging process is negligible.

**Figure 11:**
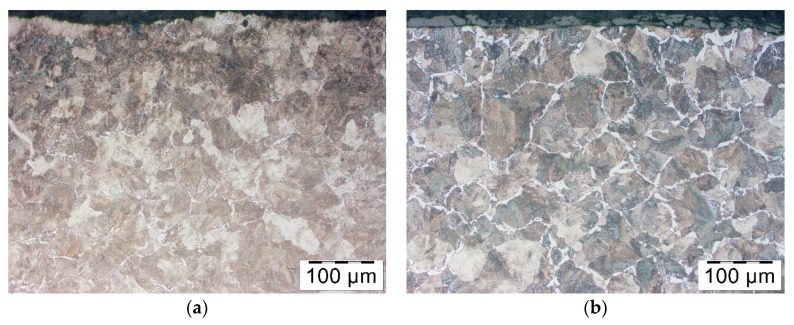
Microstructure in the surface layer of the forged part after hot forging and cooling—pre-coating of Bonderite in the concentration (**a**) 66% and (**b**) 50%.

The second substance which was tested on forged parts was Bonderite in the concentration 66% and 50%. Microstructure illustrated in Figure 11 has got relatively homogenous structure ferritic-pearlitic despite different concentrations. Only in case of the lowest concentration, in the area close to the surface of the forged part, some more prominent spots of ferrite are observable. It proves that the lubricant is successful in preventing decarburization.

**Figure 12:**
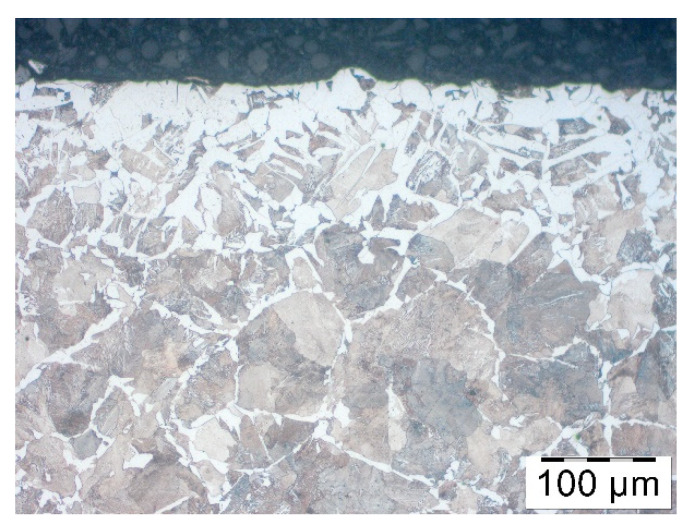
Microstructure in the surface layer of the forged part after hot forging and cooling-– pre-coating in Condursal in concentration of 100%.

The last lubricant was Condursal in the concentration of 100%. Microstructure, as shown in Figure 12, indicates that the ferrite density is high but in the distance of approximately 50 µm and gradually becomes a typical ferrite structure as seen in the deeper parts of the sample. It means that decarburization is prominent but does not go deep and is significantly weaker than in the case of a part with no coating at all.

**Figure 13:**
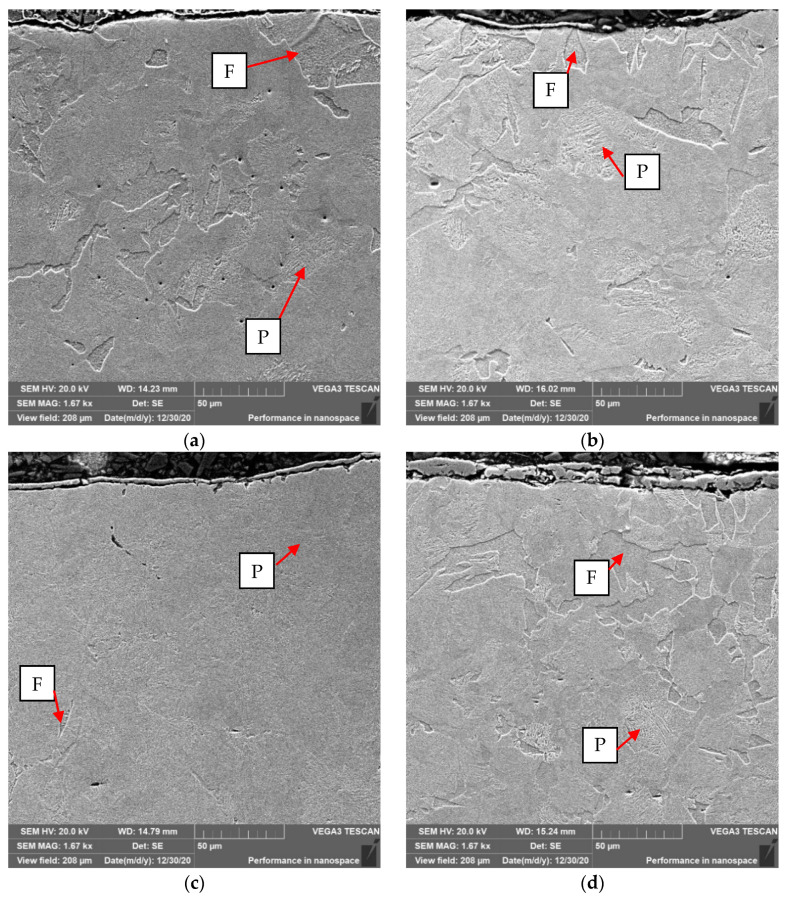
Forging surface—(**a**) without the use of protective agents, (**b**) after application of Berulit, (**c**) after application of Bonderite and (**d**) after application of Condursal.

To verify and confirm the decarburization phenomenon, SEM analysis was performed. The performed SEM microscopic analysis confirmed the tests performed on the optical microscope. A large amount of ferrite in the surface layer for unprotected forgings, for those covered with Berulite and Condursal. A slight amount of ferrite was observed in the face layer for the Bonderite coated forgings. The results of the SEM analysis are shown in Figure 13. Ferrite precipitates are marked as—F, perlite—P.

## 4. Conclusions

The tests have proved that lubrication of pre-forged parts before thermal processes may notably reduce decarburization of the surface layer of the forged parts. Under the laboratory conditions concentration of different substances was tested to see what is profitable for the rod’s lubrication and to observe how they react after drying. After warming the samples in the temperature of 1200 °C for five minutes, the researchers concluded that despite the coatings being burned out, substances like Berulite and Bonderite prevent decarburization of the surface of the bars. 

In the industrial conditions, we tested how efficient the substances were in the prevention of decarburization over the heating and forging processes. Bonderite has proved to be the leader for which no decarburization on the forged part was observed and only small-scale decarburization on the rod, which was increasing accordingly to the concentration drop.

The stages of research have also shown a correlation between the reduction of decarburization and the means of heating as well as the subsequent forging process. Generally, the rods were less decarburized than the bars, despite being heated in the same way. Apart from selecting the right lubricant, it is crucial to use the optimal concentration, taking into account the depth of decarburization and cost-effectiveness.

## Figures and Tables

**Table 1:** Atmospheres commonly used during heating.

Standard Atmospheres Produced		
Type	Composition	Application
EXO (raw)	6% combustible substances	High and low carbon steel
EXO (enhanced)	20% combustible substances	High carbon steel
Endo	60% combustible substances	Carburization

**Table 2:** Diluted atmospheres used during heating.

Diluted Atmospheres Produced		
Type	Composition	Application
Endo + Exo	15–65% combustible substances	High carbon steel
EXO (enhanced)	10–60% combustible substances	High and low carbon steel
Endo	6–20% combustible substances	High and low carbon steel

**Table 3:** Mixed atmospheres used during heating.

Mixed Atmospheres Produced		
Type	Composition	Application
Nitrogen + Methanol	8–80% combustible substances	Tool steel, High carbon steel
Nitrogen + C_n_H_m_	1–4% combustible substances	High carbon steel
Nitrogen + H_2_ + C_n_H_m_	4–12% combustible substances	High carbon steel

## Data Availability

Not applicable.
